# Platelet-Rich Fibrin Reduces IL-1β Release from Macrophages Undergoing Pyroptosis

**DOI:** 10.3390/ijms23158306

**Published:** 2022-07-27

**Authors:** Mariane Beatriz Sordi, Layla Panahipour, Zahra Kargarpour, Reinhard Gruber

**Affiliations:** 1 Department of Oral Biology, University Clinic of Dentistry, Medical University of Vienna, 1090 Vienna, Austria; mariane.sordi@kcl.ac.uk (M.B.S.); layla.panahipour@meduniwien.ac.at (L.P.); zahra.kargarpooresfahani@meduniwien.ac.at (Z.K.); 2 Department of Dentistry, Federal University of Santa Catarina, Florianopolis 88040-900, Brazil; 3 Department of Periodontology, School of Dental Medicine, University of Bern, 3012 Bern, Switzerland

**Keywords:** platelet-rich fibrin, PRF, pyroptosis, inflammasomes, periodontal diseases, macrophages

## Abstract

Background: Pyroptosis is a catabolic process relevant to periodontal disorders for which interleukin-1β (IL-1β) inflammation is central to the pathophysiology of the disease. Despite platelet-rich fibrin (PRF) anti-inflammatory properties and its application to support periodontal regeneration, the capacity of PRF to modulate pyroptosis, specifically the production and release of IL-1β, remains unknown. The question arises whether PRF could regulate IL-1β release from macrophages in vitro. Methods: To answer this question, RAW 264.7 macrophages and primary macrophages obtained from murine bone marrow were primed with PRF before being challenged by lipopolysaccharide (LPS). Cells were then analysed for the pyroptosis signalling components by gene expression analyses and IL-1β secretion at the protein level. The release of mitochondrial reactive oxygen species (ROS) was also detected. Results: PRF lowered the LPS-induced expression of IL-1β and NLRP3 inflammasome, caspase-11 and IL-18 in primary macrophages, and IL-1β and caspase-11 in RAW 264.7 cells. Additionally, PRF diminished the secretion of IL-1β at the protein level in LPS-induced RAW 264.7 cells. This was shown through immunoassays performed with the supernatant and further confirmed by analysing the lysates of permeabilised cells. Furthermore, PRF reduced the ROS release provoked by LPS in RAW 264.7 cells. Finally, to enhance IL-1β release from the LPS-primed macrophages, we introduced a second signal with adenosine triphosphate (ATP). In this setting, PRF significantly reduced IL-1β release in RAW 264.7 cells and a trend to diminish IL-1β release in primary macrophages. Conclusion: These findings suggest that PRF can reduce IL-1β release and, at least in part, inhibit pyroptosis-related factors in LPS-challenged macrophages.

## 1. Introduction

Periodontal disease is still a global health problem [1]. Following the clinical diagnosis of periodontitis or peri-implantitis, recurrently, the established treatment protocols do not successfully reach the relief of the disease to avoid tissue damage [2,3]. Therefore, primary disease prevention is of critical importance [4]. Prevention depends upon a clear understanding of the disease aetiology, in which inflammation is a central pathological mechanism causing tissue destruction [5,6,7]. Damaging signals that reach periodontal cells provoke a cascade of inflammatory events, culminating in tissue destruction [6,7,8,9]. Consequently, avoiding the exacerbation of the inflammatory cascade is probably the most suitable way to prevent periodontal disorders.

Pyroptosis is a catabolic event leading to inflammatory diseases [6,10]. Bringing knowledge from systemic inflammatory disorders to the oral environment gives insights into what clinicians know about periodontal disorders and possibilities of new approaches for periodontal therapies. Given this, pyroptosis was pointed to as one of the causes of inflammatory diseases such as rheumatoid arthritis [11,12], inflammatory bowel disease [13], and Parkinson’s disease [14], while there is increasing evidence that pyroptosis also plays an important role in periodontal diseases [6,7,15]. Pyroptosis is mainly mediated by inflammasomes, specifically the NLRP3 [16,17]. NLRP3 is closely related to the activation of caspases-1 or -11 (CAS1/CAS11). Those caspases act on the maturation of the interleukins-1β (IL-1β) and IL-18. CAS1/CAS11 also cleave gasdermin D (GSDMD), which is responsible for the cell membrane perforation and thus the release of IL-1β and IL-18 to the extracellular matrix [6,7].

IL-1β, a potent pro-inflammatory cytokine [16,18,19], promotes the loss of periodontal connective tissue and bone [20]. Clinically, high levels of IL-1β were detected in the gingival crevicular fluid of periodontitis patients [21,22]. In vivo, IL-1β deficient mice demonstrated less LPS-induced periodontium destruction than wild-type mice undergoing the same treatment [20], supporting IL-1β as a pathologic factor driving catabolic processes. However, it is unclear if the IL-1β action is a consequence of pyroptotic cell membrane perforation. In vitro, IL-1β is not released until LPS-primed macrophage membrane perforation is induced by adenosine triphosphate (ATP) and other components [16,18]. Thus, the IL-1β release into the cell supernatant is a hallmark for pyroptosis-mediated membrane perforation as it might occur in periodontal disorders.

Platelet-rich fibrin (PRF) is a current approach to preventing or reducing tissue inflammation. There are different routes to obtain a variety of PRFs, for instance, pure PRF (P-PRF), leukocyte-PRF (L-PRF), and injectable PRF (I-PRF) for the use in clinical dentistry [22]. At the cellular level, our group already showed the potential of PRF to decrease the pro-inflammatory response of macrophages against LPS by reducing IL-1β [23], IL-6 [23,24], and cyclooxygenase-2 (COX2) [24]. PRF also decreased the expression of IL-6 and nitric oxide synthase (iNOS) in bone marrow-derived ST2 cells and 3T3-L1 mesenchymal cells, and reduced the phosphorylation and nuclear translocation of p65 in ST2 cells [25]. Remarkably, PRF even eliminated the hydrogen-peroxide-induced toxicity in the gingival fibroblasts [26]. Thus, there is a robust suggestion for applying PRF to reduce inflammatory reactions in vitro. However, the capacity of PRF to modulate pyroptosis, specifically the production and the release of IL-1β, remains unknown.

There is strong evidence that PRF has anti-inflammatory properties [23,24,25,27] and pyroptosis is linked to the activation of the inflammasome and release of IL-1β in periodontal disorders [6,7,15]. Thus, the question arises whether PRF could reduce pyroptosis factors in macrophages, which are closely related to inflammatory reactions. Specifically, we focused on the release of IL-1β, a hallmark for inflammation that is closely related to pyroptosis. Therefore, we tested the hypothesis that the anti-inflammatory activity of PRF—at least partially—involves a lowering of the secretion of maturated IL-1β in LPS-challenged macrophages.

## 2. Results

### 2.1. PRF Reduces the Expression of CAS11 and IL-1β in LPS-Induced RAW 264.7 Macrophages

**Figure 1:**
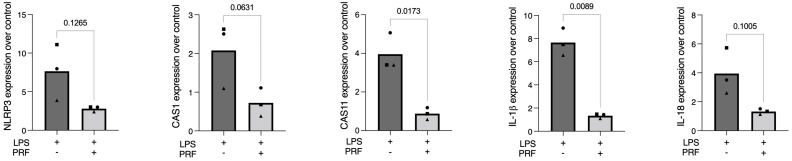
PRF reduces the expression of pyroptosis-related genes in LPS-induced RAW 264.7 macrophages. A significant reduction in CAS11 and IL-1β in LPS-induced RAW 264.7 macrophages was reported. Gene expression is compared to the untreated control, and each data point represents an independent experiment. *n* = 3. To compare groups, paired *t*-test was applied.

To establish a model on the gene expression of the pyroptosis-related factors, RAW 264.7 macrophages were exposed to LPS and followed by the screening for the respective marker genes. Concerning pyroptosis, NLRP3, CAS1, and CAS11, the central components of the canonical pathway of pyroptosis, were increasingly expressed in the LPS-exposed RAW 264.7 macrophages, which was reduced in the presence of PRF. We further report that PRF significantly lowered the forced expression of IL-1β (Figure 1); however, the expression of the selected pyroptosis-related interleukins was relatively low. Similar finding were reported regarding IL-18.

### 2.2. PRF Reduces the Expression of NLRP3, CAS11, IL-1β, and IL-18 in LPS-Induced Primary Macrophages

**Figure 2:**
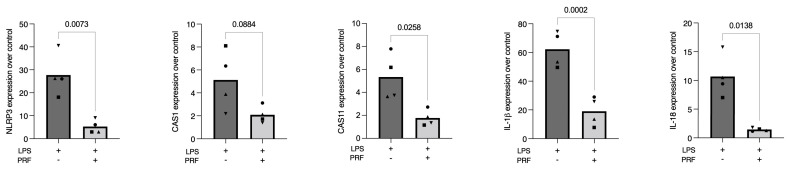
PRF reduces the expression of pyroptosis-related genes in LPS-induced primary macrophages. A significant reduction in NLRP3, CAS11, IL-1β, and IL-18 in LPS-challenged primary macrophages was reported. Gene expression is compared to the untreated control, and each data point represents an independent experiment. *n* = 4. To compare groups, paired *t*-test was applied.

To verify the findings on pyroptosis-related factors regarding gene expression, we used primary macrophages obtained from the bone marrow of mice. Cells were then exposed to LPS and after priming with PRF. In this setting, primary macrophages showed the expected increased expression of the inflammasome NLRP3, the caspases CAS1 and CAS11, and the lead cytokines IL-1β and IL-18. We found that PRF significantly lowered the forced expression of NLRP3, CAS11, IL-1β, and IL-18 in LPS-induced primary macrophages (Figure 2). In the setting, however, a higher fold-change expression of IL-1β secretion was found, suggesting that maybe the primary macrophages are more susceptible to LPS challenges regarding pyroptosis and cell disruption.

### 2.3. PRF Decreases the Reactive Oxygen Species (ROS) Release in LPS-Induced RAW 264.7 Macrophages

**Figure 3:**
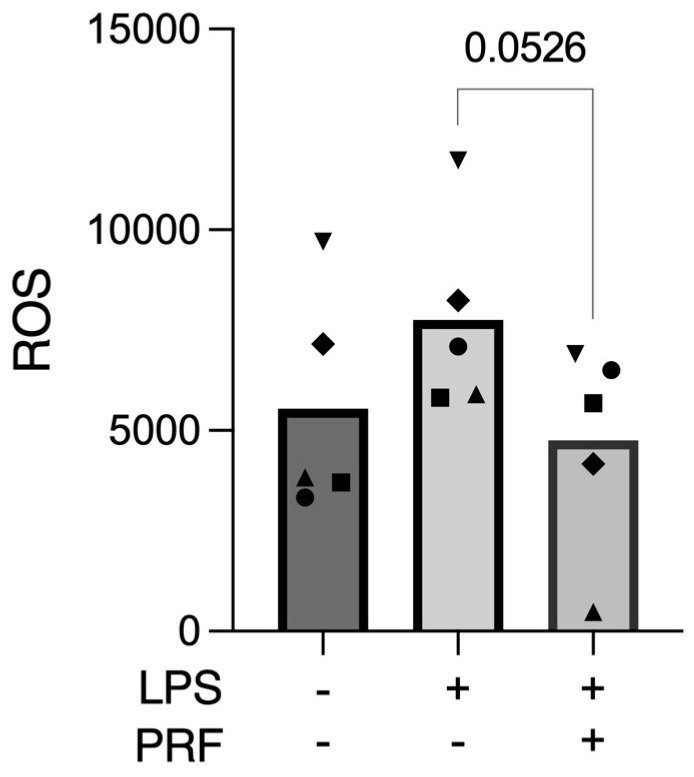
PRF reduced the ROS release provoked by LPS in activated RAW 264.7 macrophages. *n* = 5. To compare groups, paired *t*-test was applied.

ROS production occurs after K^+^ deprivation [28,29] and acts downstream of gene transcription, mRNA translation, and IL-1β converting enzyme activation [29], which can thus induce pyroptosis [6]. Therefore, to assess the cell stress towards pyroptosis caused by the LPS stimulus, we evaluated the mitochondrial ROS release. Here, we report that PRF decreased the ROS release provoked by LPS on RAW 264.7 macrophages (Figure 3).

### 2.4. PRF Might Exert the Same Activity on Macrophages’ Release of IL-1β as Pyroptosis-Specific Inhibitors

**Figure 4:**
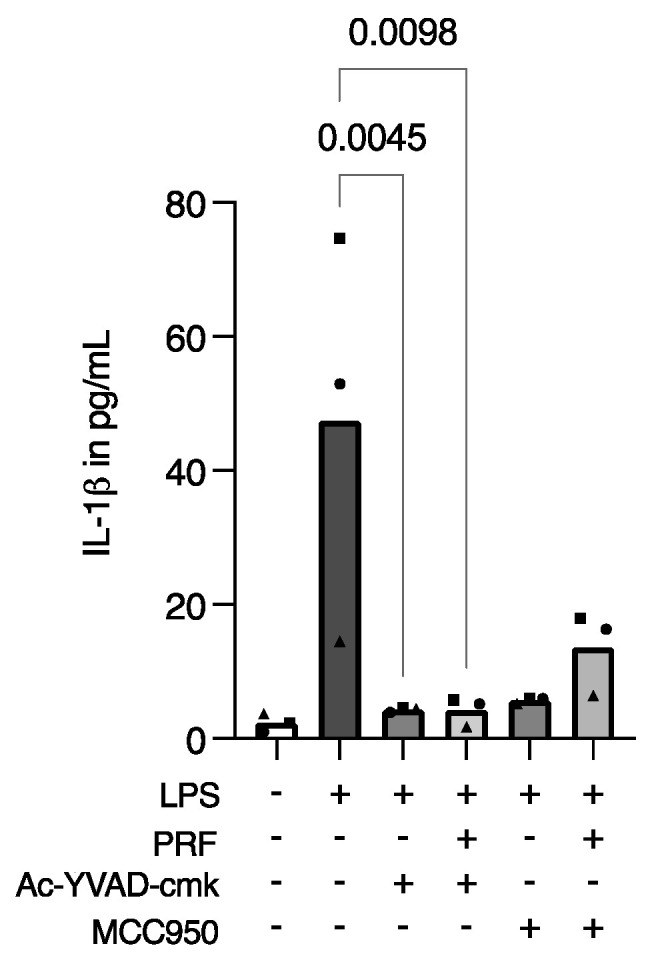
PRF exerts similar activity to pyroptosis-specific inhibitors regarding the release of IL-1β in LPS-induced RAW 264.7 macrophages. A reduction in IL-1β in LPS-induced RAW 264.7 macrophages was reported after cell stimulation with Ac-YVAD-cmk and MCC950, in the presence or not of PRF in RAW 264.7 macrophages. Each data point represents an independent experiment. *n* = 3. To compare groups, the Friedman test followed by Dunn’s multi-comparison test was applied.

To determine if PRF is exerting activity on the pyroptosis pathway of inflammation, we introduced two specific pyroptosis inhibitors. Then, Ac-YVAD-cmk, a caspase-1 inhibitor, and MCC950, an NLRP3 inhibitor, were applied alone or in combination with PRF in LPS-stimulated RAW 264.7 cells, and IL-1β immunoassays were performed. As expected, LPS increased IL-1β release, which was further decreased in the presence of Ac-YVAD-cmk and MCC950, either with or without PRF stimulation (Figure 4). These findings indirectly suggest that the PRF has the same activity as those inhibitors on pyroptosis in an in vitro macrophage model.

### 2.5. PRF Diminishes the Release of IL-1β in LPS-Induced RAW 264.7 Macrophages

**Figure 5:**
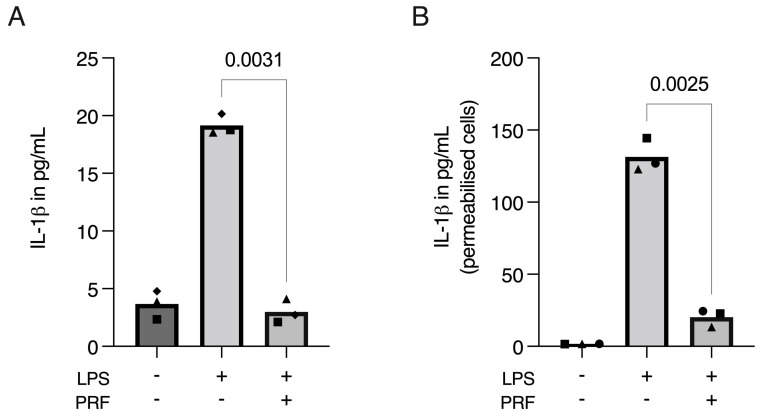
PRF moderates the release of IL-1β in LPS-induced RAW 264.7 macrophages. A significant reduction in IL-1β in LPS-induced RAW 264.7 macrophages was found on cell supernatants (**A**), which was boosted and reproduced when the cell membranes were permeabilised with Triton X-100 (**B**), showing that IL-1β was produced, but not released, to the extracellular media following the LPS challenging on RAW 264.7 macrophages. Each data point represents an independent experiment. *n* = 3. To compare groups, paired *t*-test was applied.

Considering PRF has anti-inflammatory properties [23,24,25,26,27], and pyroptosis is related to the production and release of IL-1β [6,9,30,31], we further aimed to determine if PRF could diminish the release of IL-1β. The collected supernatant was then used for IL-1β immunoassay, where we found a reduction in IL-1β in LPS-induced RAW 264.7 macrophages (Figure 5A). However, LPS-primed RAW 264.7 cells did not show an intense release of IL-1β. Therefore, to evaluate if the cells were producing—but not releasing—IL-1β, we disrupted the cell membranes with Triton X-100 and again performed an IL-1β immunoassay. Using a detergent significantly increased IL-1β in the supernatant of LPS-induced RAW 264.7 macrophages and further supported the anti-inflammatory activity of PRF (Figure 5B).

### 2.6. PRF Reduces the Release of IL-1β in ATP-Induced, LPS-Primed RAW 264.7 Cells and Primary Macrophages

**Figure 6:**
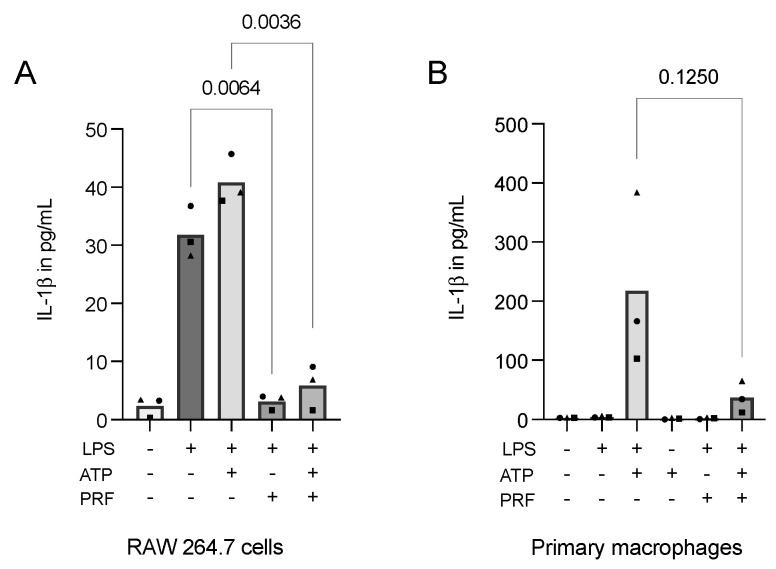
PRF reduces the secretion of IL-1β in ATP-induced, LPS-primed RAW 264.7 cells (**A**) and primary macrophages (**B**). A reduction in IL-1β following the introduction of PRF in RAW 264.7 cells and primary macrophages that were stimulated with LPS and received a second signal from ATP was reported. Each data point represents an independent experiment. *n* = 3. To compare groups, individually paired *t*-tests were applied.

Considering the obtained data, we might conclude that LPS alone could not fully provoke pyroptosis on macrophages in vitro. Moreover, pondering the finding that cell permeabilization boosts IL-1β detection in immunoassays, we introduced ATP, which is known for its potential to increase IL-1β release as a second signal for cells previously challenged with LPS [18,32]. In RAW 264.7 macrophages, ATP increased the secretion of IL-1β compared to LPS alone. Importantly, PRF was able to significantly reduce the interleukin secretion in both applied settings (Figure 6A). These findings were even more pronounced in primary macrophages (Figure 6B).

Lastly, to investigate if PRF could rescue LPS-primed, ATP-induced RAW 264.7 macrophages, we introduced PRF later in the in vitro model. PRF could not exert the same role in rescuing cells from the damage, as the release of IL-1β was not significantly reduced in the presence of PRF (Supplement Figure S1), and cell viability was not reversed with later the application of PRF (Supplement Figure S2). In addition, we report that ATP alone is not able to produce and release IL-1β in RAW 264.7 cells.

## 3. Discussion

Pyroptosis is a caspase-dependent catabolic process in which IL-1β is a powerful pro-inflammatory cytokine responsible for tissue damage in inflammatory disorders [6,10,18,32]. Despite PRF’s anti-inflammatory properties [23,24,25,26,27] and its successful application to support periodontal regeneration [33,34,35], its capacity to modulate pyroptosis through the analyses of IL-1β remains uncertain. At the transcriptional level, PRF lowered the LPS-induced expression of IL-1β, NLRP3, CAS11, and IL-18 in primary macrophages and IL-1β and CAS11 in RAW 264.7 cells. Additionally, PRF reduced the ROS release provoked by LPS-activated RAW 264.7 cells. Then, we sought to consider if PRF could regulate IL-1β release from macrophages, the inflammatory cells from the immune system, in vitro. We report that PRF diminished the release of IL-1β at the protein level in LPS-induced RAW 264.7 cells. This was shown through immunoassays performed with the cell supernatants and further confirmed by analysing the lysates of cells permeabilised with Triton X-100. Finally, to enhance IL-1β release from the LPS-primed RAW 264.7 cells, we introduced a second signal with ATP. Again, PRF significantly reduced IL-1β release in RAW 264.7 macrophages and tended to diminish IL-1β release in primary macrophages.

The ROS release results confirm that RAW 264.7 cells are suffering from the challenge of LPS alone, even if LPS is not toxic to the cells. We also speculated, by applying the LPS-stimulated RAW 264.7 cells and IL-1β detection, the possible pathway of PRF over the cells to provoke such results. Then, we applied the Ac-YVAD-cmk caspase-1 inhibitor and the MCC950 inflammasome inhibitor, in combination or not with PRF, on LPS-stimulated cells. A significant reduction in IL-1β detection under Ac-YVAD-cmk or Ac-YVAD-cmk + PRF stimulation was found for LPS-challenged RAW 264.7 cells. Similarly, a trend in reducing IL-1β levels under MCC950 or MCC950 + PRF stimulation in LPS-challenged RAW 264.7 cells was reported. This suggests that PRF might be acting preferably by blocking CAS1 instead of primary blocking of the inflammasome assembly. This is an interesting finding; however, we cannot assure the exact pathway PRF exerts on macrophages in vitro.

In support of existing knowledge [18,32], we show that LPS alone could not provoke the full cascade of pyroptosis on macrophages. This was verified by the weak expression of NLRP3 inflammasome and CAS1/CAS11, especially in RAW 264.7 macrophages, while primary macrophages responded with a stronger expression of the pyroptosis-related genes under LPS stimulation. In addition, at the protein level, CAS1 and IL-1β showed low expression levels. The maturation of IL-1β by caspases should be followed by its release from the pores formed in the cell membrane, which is the role of pyroptosis executor GSDMD [28,36]. To evaluate if our cells were producing but not releasing IL-1β, we added Triton X-100, a commonly used detergent for cell membrane permeabilization, to the cells and analysed the collected lysates. Then, we could observe that the protein levels of IL-1β increased about seven times while the information remained the same, i.e., PRF significantly reduced the IL-1β production by LPS-stimulated RAW 264.7 cells, suggesting that the matured IL-1β was mainly trapped inside the cells.

While ATP is related to the strong increase in the IL-1β release in the LPS-primed macrophages [18,32], other compounds were indicated to play important roles in the pyroptosis pathway, such as α-hemolysin [37,38,39] and nigericin [40,41], either alone or in combination with other pathogen-associated molecular patterns (PAMPs). Alpha-hemolysin is a bacterial pore-forming toxin produced by *Staphylococcus*, which activates inflammasome activity and caspase-1, thus inducing pyroptosis [38,42]. Nigericin is a microbial toxin produced by *Streptomyces hygroscopicus* that decreases intracellular potassium, which causes caspase-1 activation and induces the release of IL-1β, leading to pyroptosis [30,43]. Studies on macrophages have been trying to find molecules to reduce pyroptosis in LPS-primed ATP/α-hemolysin/nigericin-stimulated cells [41] or even exacerbate pyroptosis cell death due to the potential application in anti-infection or anti-tumour immunity [40]. Nevertheless, this topic is relatively new in periodontal research.

When stimulated with PAMPs, such as LPS, cells produce inactive pro-IL-1β with a molecular weight of 31 kDa, which accumulates in the cytosol [31,44]. When a second signal, such as ATP, activates the cation-selective P2X7 receptor on the cell membrane [18], a potassium efflux occurs via a pore permeable to hydrophilic solutes up to 900 Da [45,46,47]. This drop in intracellular potassium triggers the assembly of the inflammasome protein complex, leading to the production of active CAS1. In turn, activated CAS1 cleaves pro-IL-1β into the active 17 kDa form, which is then released to the extracellular media [31,32]. Therefore, the initial priming affects NLRP3 and pro-IL-1β at the mRNA transcription level, and the second signal mediates the assembly of the inflammasome, resulting in CAS1 activation and release of IL-1β [16]. Accordingly, with this setting, the total levels of IL-1β are higher, but intracellular pro-IL-1β levels remain similar when comparing the LPS and LPS + ATP stimulation [18]. Thus, the application of ATP provokes cell membrane pore formation and the release of intracellular IL-1β, similar to what is expected with the application of the detergent Triton X-100 that we also used herein.

The question then arises if PRF protects the cell membrane from ATP-induced disruption. Therefore, we performed nuclear staining that indicates the integrity of the cell membrane. Indeed, ATP permeabilised the membrane of RAW 264.7 macrophages, as indicated by the red staining of the nuclei (Supplement Figure S2); however, that pattern was not changed in the presence of PRF, thus suggesting that PRF cannot protect the cells from ATP-induced membrane disruption. The lower IL-1β release is likely a consequence of the reduction in the transcription level rather than a lowering of the ATP-induced membrane permeabilization.

Our study limitations comprise the limitations of any in vitro study. As such, we cannot extend the findings of the present research to a clinical aspect. Nevertheless, the use of PRF is well-established in the clinical application, and the findings that PRF diminishes IL-1β release in LPS- or LPS + ATP-stimulated cells support the continuous use of blood-derived strategies as a therapy for periodontal complications or regenerative procedures. In this sense, other forms of PRF production, such as liquid PRF, which consists of large liquid platelet-poor plasma (PPP) layer that is almost devoid of cells, and the buffy coat layer accumulating the platelets and leucocytes termed concentrated PRF or C-PRF [24], could be further tested. Notably, the distribution and concentration of fibrinogen are different among the several fractions of liquid PRF [48], and it may impact the anti-inflammatory activity. Further studies on pyroptosis, however, should focus on LPS + ATP-stimulated cells, a model that is able to provoke the full cascade of pyroptosis in macrophages. Even though we have decided to focus on macrophages, the central cells regarding inflammatory processes, other periodontal-related cells, such as oral epithelial cells and gingival fibroblasts, should be used as a target for LPS priming followed by ATP stimulation in future studies.

Given the data obtained, we conclude that PRF can reduce IL-1β release and, at least partially, inhibit pyroptosis-related factors in LPS-induced macrophages in vitro. We can further recommend the continuous clinical application of PRF for periodontal therapy since our in vitro findings support the well-established anti-inflammatory role of PRF.

## 4. Materials and Methods

### 4.1. Platelet-Rich Fibrin (PRF) Preparation

PRF lysates were prepared following the established protocol of our group [23]. PRF was prepared after acquiring the approval of the Ethics Committee of the Medical University of Vienna (1644/2018), and volunteers signed informed consent. All experiments were performed in accordance with relevant guidelines and regulations at the Medical University of Vienna. Briefly, venous blood was collected from five healthy volunteers using 10 mL glass tubes with no silica/silicon treatment (Bio-PRF, Venice, FL, USA). Then, PRF membranes were produced by centrifugation at 700× *g* for 8 min (Bio-PRF, Venice, FL, USA). The PRF clot was separated from the remaining red thrombus and squeezed out using a pressing plate (Bio-PRF, Venice, FL, USA). Each PRF membrane was transferred into a serum-free medium (1 cm PRF/mL; DMEM; Sigma Aldrich, St. Louis, MO, USA) and exposed to two cycles of freeze-thawing and sonication to produce the PRF lysates. After centrifugation at 15,000× *g* for 10 min, the lysates were sterile-filtered with a 0.2-micron mesh Millipore filter and stored at −80 °C before the in vitro analysis.

### 4.2. Primary Macrophages and RAW 264.7 Macrophage-Like Cells

BALB/c mice of 6 to 8 weeks old were purchased from Animal Research Laboratories, Himberg, Austria. Bone marrow cells were collected from the femora and tibiae as previously described [49]. Bone marrow cells were seeded at 1 × 10^6^ cells/cm^2^ into 24-well plates and grown for 7 days in Dulbecco’s Modified Essential Medium (DMEM; Sigma Aldrich, St. Louis, MO, USA) supplemented with 10% foetal calf serum (FCS; Capricorn Scientific GmbH, Ebsdorfergrund, Germany), 1% antibiotics (PS; Sigma Aldrich, St. Louis, MO, USA), and 20 ng/mL macrophage colony-stimulating factor (M-CSF; ProSpec, Ness-Ziona, Israel). Primary macrophages are closer to the in vivo situation, and therefore, the obtained cells were used to carry out selected experiments to confirm our hypothesis. However, the use of primary macrophages requires the sacrifice of mice. For this reason, we performed additional experiments using a cell line to reduce and replace animal organ donation. Thus, RAW 264.7 macrophage-like cells (LGC Standards, Wesel, Germany) were expanded in a growth medium and seeded at 3 × 10^5^ cells/cm^2^ into 24-well plates. Cells were primed with 30% PRF in serum-free media for 1 h and then exposed to 100 ng/mL of LPS from Escherichia coli 055:B5 (Sigma Aldrich, St. Louis, MO, USA) for 6 h to induce an inflammatory response. Pyroptosis-specific inhibitors were applied to establish the in vitro LPS-induced pyroptosis model for PRF testing. MCC950 (CP-456773, Selleck Chemicals GmbH, Houston, TX, USA), an inflammasome NLRP3 inhibitor, was applied at 8.0 µM for 30 min before cells were exposed to LPS. Ac-YVAD-cmk (≥95%, HPLC, Sigma Aldrich, St. Louis, MO, USA), a caspase-1 inhibitor, was applied at 5.0 µM for 20 h prior LPS challenge. Alternatively, to increase IL-1β protein release from LPS-primed cells, ATP (InvivoGen, San Diego, CA, USA), an NLRP3 inflammasome inducer, was introduced at 5.0 mM for 1 h in LPS-primed cells. All cell lineages were exposed to the treatments under standard conditions at 37 °C, 5% CO_2_, and 95% humidity.

### 4.3. Reverse Transcription Quantitative Real-Time PCR (RT-qPCR)

For RT-qPCR, after stimulation, total RNA was isolated with the ExtractMe total RNA kit (Blirt S.A., Gdańsk, Poland), followed by reverse transcription and polymerase chain reaction (LabQ, Labconsulting, Vienna, Austria) on a CFX Connect™ Real-Time PCR Detection System (Bio-Rad Laboratories, Hercules, CA, USA). The mRNA levels were calculated by normalizing to the housekeeping gene GAPDH using the ^ΔΔ^Ct method. The primer sequences were NLRP3-F: TCACAACTCGCCCAAGGAGGAA; NLRP3-R: AAGAGACCACGGCAGAAGCTAG; CAS1-F: GGCACATTTCCAGGACTGACTG; CAS1-R: GCAAGACGTGTACGAGTGGTTG; CAS11-F: CCTGAAGAGTTCACAAGGCTT; CAS11-R: CCTTTCGTGTAGGGCCATTG; GSDMD-F: GGTGCTTGACTCTGGAGAACTG; GSDMD-R: GCTGCTTTGACAGCACCGTTGT; IL-1β-F: CAACCAACAAGTGATATTCTCCATG; IL-1β-R: GATCCACACTCTCCAGCTGCA; IL-18-F: CAAACCTTCCAAATCACTTCCT; IL-18-R: TCCTTGAAGTTGACGCAAGA; GAPDH-F: AACTTTGGCATTGTGGAAGG; and GAPDH-R: GGATGCAGGGATGATGTTCT. RT-PCR data are represented compared to the untreated control.

### 4.4. Immunoassays

For immunoassays, supernatants were collected from the stimulated cells and were analysed for IL-1β release (R&D Systems, Minneapolis, MN, USA) according to the manufacturer’s instruction. Since the release of IL-1β from the supernatant was relatively low, we prepared cell lysates with 0.3% Triton X-100 (Sigma Aldrich, St. Louis, MO, USA) and collected the lysates for IL-1β immunoassay under the same conditions.

### 4.5. Mitochondrial Reactive Oxygen Species (ROS) Release

RAW 264.7 cells were seeded at 3 × 10^5^ cells/cm^2^ into 96-well plates. On the next day, cells followed the standard stimulation with 30% PRF and were then challenged with 100 ng/mL LPS for 6 h. Cells were analysed for the release of mitochondrial reactive oxygen species (MitoROS^TM^ 580, AAT Bioquest, Inc., Sunnyvale, CA, USA) according to the manufacturer’s instructions.

### 4.6. Statistical Analysis

All experiments were performed at least three times. Statistical analyses were performed with paired *t*-tests or Friedman tests comparing the relevant groups. Analyses were performed using Prism v.9 (GraphPad Software, La Jolla, CA, USA). Significance was set at *p* < 0.05.

## Data Availability

Raw data are made available on request.

## Supplementary Materials

The following supporting information can be downloaded at: https://www.mdpi.com/article/10.3390/ijms23158306/s1.
