# Supplementary material for: Platelet-Rich Fibrin Reduces IL-1β Release from Macrophages Undergoing Pyroptosis
Source: Int J Mol Sci. 2022 Jul 27;23(15):8306. doi: 10.3390/ijms23158306 (PMC9368224; doi:10.3390/ijms23158306)
Supplement: Supplementary file 1 [file ijms-23-08306-s001.zip › ijms-1809219-supplementary.pdf]

Supplement Files

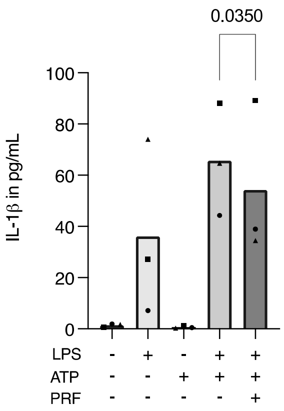

Figure S1. PRF does not reduce the secretion of IL-1β in ATP-induced, LPS-primed RAW 264.7 cells when PRF is introduced later in the bioassay. The further increase of IL-1β following the introduction of ATP in RAW 264.7 cells was not reduced in the presence of PRF when it was introduced after priming the cells with LPS. Each data point represents an independent experiment. N = 3. To compare groups, individual paired t-tests were applied.

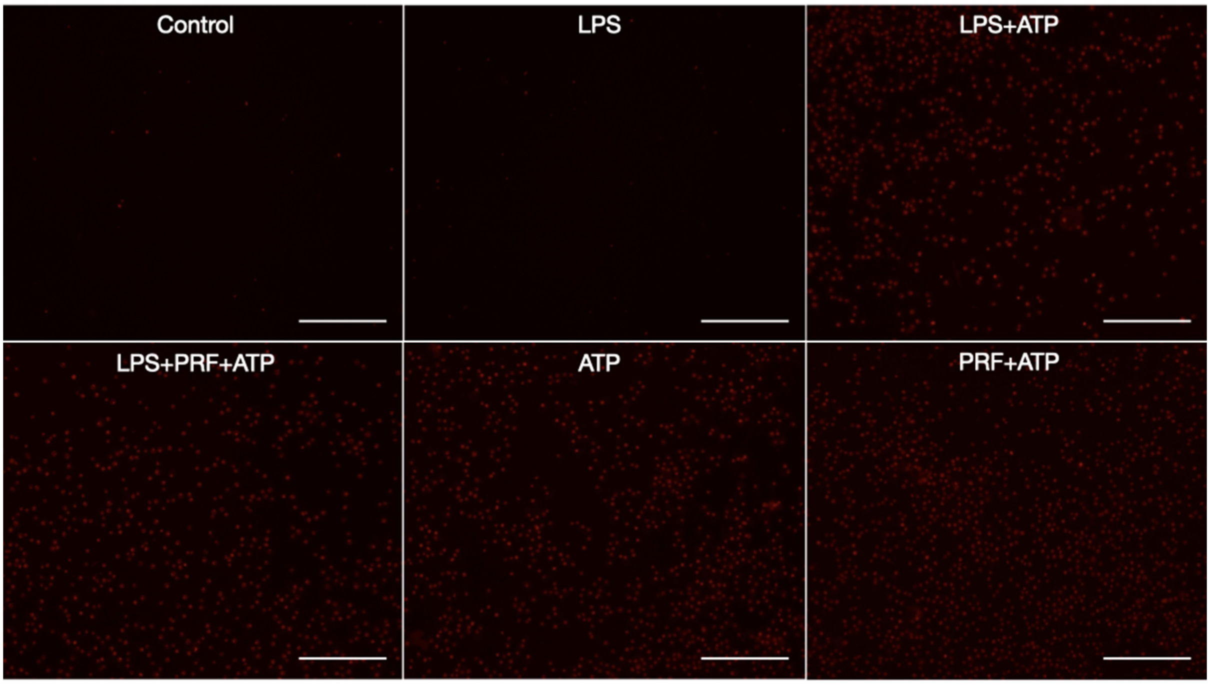

Figure S2. PRF does not rescue cells from the ATP-induced damage, as shown by the Live-dead assay in stimulated RAW 264.7 macrophages. Scale bars represent 100 µm.
